# Liquid Resin Infusion Process Validation through Fiber Optic Sensor Technology

**DOI:** 10.3390/s22020508

**Published:** 2022-01-10

**Authors:** Vincenzo Romano Marrazzo, Armando Laudati, Michele Vitale, Francesco Fienga, Gianni Iagulli, Marco Raffone, Andrea Cusano, Michele Giordano, Antonello Cutolo, Giovanni Breglio

**Affiliations:** 1 Department of Electrical Engineering and Information Technology (DIETI), University of Naples “Federico II”, 80125 Naples, Italy; francesco.fienga@unina.it (F.F.); antonello.cutolo@unina.it (A.C.); breglio@unina.it (G.B.); 2 OptoSmart S.R.L., 80121 Naples, Italy; a.laudati@optosmart.com (A.L.); m.vitale@optosmart.com (M.V.); a.cusano@unisannio.it (A.C.); gmichele@unina.it (M.G.); 3 Leonardo S.P.A.—Aircraft Division, Viale dell’Aeronautica, 80038 Naples, Italy; gianni.iagulli@leonardocompany.com (G.I.); marco.raffone@leonardocompany.com (M.R.); 4 Department of Engineering, University of Sannio, Corso Garibaldi, 82100 Benevento, Italy; 5 Institute of Composite Materials Technology National Research Council (ITMC-CNR), 80125 Naples, Italy

**Keywords:** fiber optic sensor, fiber Bragg grating, sensor network, temperature measurement, liquid resin infusion, composite material

## Abstract

In the proposed work, a fiber-optic-based sensor network was employed for the monitoring of the liquid resin infusion process. The item under test was a panel composed by a skin and four stringers, sensorized in such a way that both the temperature and the resin arrival could be monitored. The network was arranged with 18 Fiber Bragg Gratings (FBGs) working as temperature sensors and 22 fiber optic probes with a modified front-end in order to detect the resin presence. After an in-depth study to find a better solution to install the sensors without affecting the measurements, the system was investigated using a commercial Micron Optics at 0.5 Hz, with a passive split-box connected in order to be able to sense all the sensors simultaneously. The obtained results in terms of resin arrival detection at different locations and the relative temperature trend allowed us to validate an infusion process numerical model, giving us better understanding of what the actual resin flow was and the time needed to dry preform filling during the infusion process.

## 1. Introduction

As in most cases in the engineering field, manufacturing methods are driven principally by the cost and the quality of the purpose, subjected to a trade-off. An example is given for the improvement of the manufacturing process of large-area structures in composite material, which is centered on Resin Transfer Molding (RTM, as described in [1]), compression and autoclave vacuum bag methods. RTM is very popular since is possible to manufacture good-quality composite parts with a fast infusion, providing an optimal ending surface. Nevertheless, many disadvantages exist in terms of cost, the use of the correct metal tools and geometric limitations [2]. Moreover, the more is the time needed to infuse the resin, the higher the cost and complexity will be. In this context, the liquid resin infusion (LRI) has the potential to mitigate the limitation presented by RTM [3,4] in order to: (i) improve the quality and the reliability; (ii) reduce the time processes; (iii) reduce the fabrication costs; (iv) reduce the environmental impact of the process; and (v) increase the range of geometrical complexity and the dimensions of infused parts. An example of LRI is Vacuum-Assisted Resin Transfer Molding (VARTM), described in [5], which facilitates the fabrication of large parts. Since composite manufacturing is performed in an oven, a predictive simulation of LRI infusion would be very helpful and is, in the state-of-the-art literature, the subject of a number of publications [6,7,8]. For the RTM technique, analysis code and Finite Element tools were designed as described in [9,10,11,12]. LRI analysis can be performed using similar techniques used for RTM, with many changes in fiber volume ratio, porosity and the application of Darcy’s law [13], which describes the flow of a fluid through a porous medium. The abovementioned changes involve a more complex iterative solution for the simulator; solutions for this class of problem are mentioned in [14,15,16].

The main problem, in the framework of manufacturing using LRI methods, is because the latter relies on a “trial and error” approach to enable the final setup process to be correct. Certainly, the monitoring of resin flow can help the operator but, since the infusion is conducted in an oven, real-time visual monitoring is impossible. In these cases, the only information available to the operator may be that given by a series of sensors placed strategically to monitor the flow, giving the operator confidence in the completion of infusion.

This paper presents the monitoring of temperature and resin arrival in the LRI process employing Fiber Optic Sensor (FOS) technology, with the aim to validate a pre-existent numerical model. A simulation model is typically used for LRI process optimization in terms of the infusion strategy (the number and position of resin inlets and outlets) and bagging scheme of new parts by reducing the number of manufacturing trials needed for the development of the process. The choice to use FOS technology in order to realize the required monitoring system presents several advantages, compared to the traditional sensors, for temperature measurements above all. The main advantages are: (i) immunity to electromagnetic interference; (ii) few losses during the signal propagation; (iii) high information transmittivity; (iv) the possibility for multichannel communication; (v) a long distance between the system and the sensors; (vi) passive elements; (vii) on the same optical fiber, a larger number of sensors can be installed, reducing the overall number of cables; and (viii) it is suitable for harsh environments, as demonstrated in [17]. Thanks to its limited dimensions, a FOS can easily be embedded in composite materials without creating defects in the solid laminate. Among the various types of FOSs, the natural candidate to achieve the aim of this work was the Fiber Bragg Grating (FBG) sensor, which was employed for the monitoring of the temperature while, for resin detection, a modified front-end of the optical fiber was designed.

## 2. Problem Definition

With the aim to investigate and consolidate a fabrication method for carbon fiber panels produced with the LRI process, a fiber-optic-based sensor network installed on a dried preform was designed. To better improve the reliability of the infusion system, resin discharge pipes were sensorized as well.

**Figure 1:**
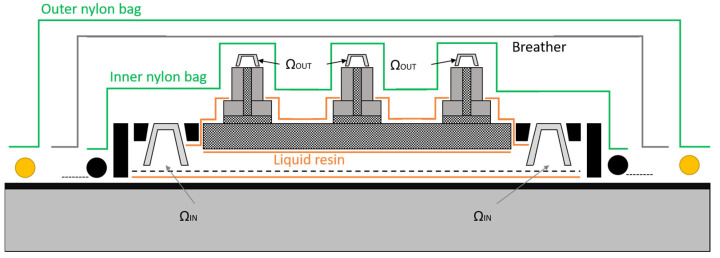
Cross-section of a generic panel “stringerized” and prepared for the infusion.

The item that was sensorized was a rectangular panel of 1 m × 0.6 m composed of a flat skin with 2.4 mm thickness, reinforced with four stringers of 0.9 m × 0.06 m with a thickness of 2.8 mm. The latter, since it needed to be pre-heated at a given temperature to allow the resin to flow correctly, inhaling the vacuum from the output pipes, was realized in an autoclave/oven. For the purpose of the case study, it is important to define a better configuration for the sensor installation in terms of the points of interest along the item and the pipes; in Figure 1, a cross-section of the “skin” and the “stringer” of a generic panel prepared for the infusion process is displayed. It is also possible to note the type of “auxiliary” material needed for the process.

**Figure 2:**
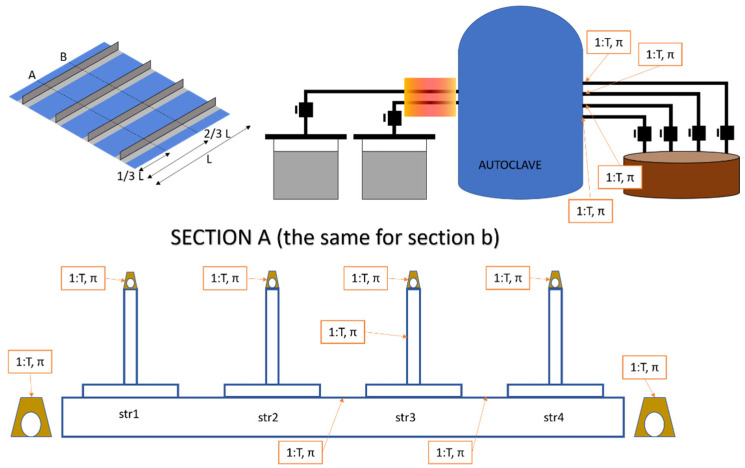
Comprehensive scheme of the sensorized panel and the infusion plant.

The purpose of this work was to monitor the temperature value (*T*) and the resin presence (π) in input pipes, noted as $\Omega_{IN}$, in output pipes noted as $\Omega_{OUT}$, as well as in specific locations inside and over the panel. In order to better understand the positioning used in the FOS method, a descriptive table (Table 1) and the relative representation (Figure 2) follows.

**Figure 3:**
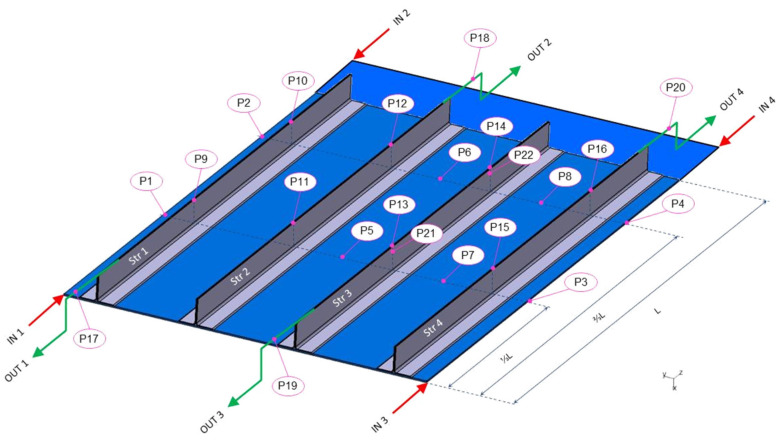
Illustration of the effective position of the whole fiber optic sensor network.

From the previous table and figure, it can be noted that a total of 22 sensors were employed to monitor π, and 18 to monitor T (as represented in Figure 3). After these considerations, the requirements for the monitoring system were:

- For each T, a π probe was associated.
- Regarding the 22 points, 18 were both T and π.
- Acquisition frequency was in the order of Hz (static measurement).
- The thickness of the FOS was minimized.

Taking the abovementioned into account, the fiber optics employed were characterized by a polyimide coating, which gave them a very low thickness (155 μm) with high mechanical resistance. Regarding the fiber optics used as connections, a further protective jacket of PTFE with 1 mm thickness was used to increase the robustness.

### 2.1. π Probe for Resin Detection

For this kind of detection, it is necessary to create interaction between the optical radiation, confined within the optical fiber, and the external environment. Many methods exist in the literature, as well as in commerce. For the described application, the simplest and most cost-effective solution is to design a front-end whose reflectivity is modulated by the density of the external medium, that is the resin or the air. From the Fresnel law, it is noted that:

(1) $$R={\left[\frac{n_{f}-n_{m}}{n_{f}+n_{m}}\right]}^{2}$$

where *R* is the reflection coefficient between the fiber optic, with refraction index $n_{f}$, and the external medium, with refraction index $n_{m}$. For a Single-Mode Fiber (SMF), $n_{f}=1.47$ at 1550 nm, while $n_{m\;air}=1$ and $n_{m\;resin}=1.5$. From the equation written above, $R_{air}=0.036$, while $R_{resin}=0.0001$. Thus, the two different mediums imply a change in R which differs from 3% in the case of air to 0.01% in the case of resin. Since the reflectivity is relevant, the detection method was easily based on the injection of optical power inside the fiber containing the sensor, continuously detecting the reflected part. Therefore, the π probe was manufactured by orthogonally cutting a fiber optic (in polyimide coating) with a dedicated precision tool.

**Figure 4:**
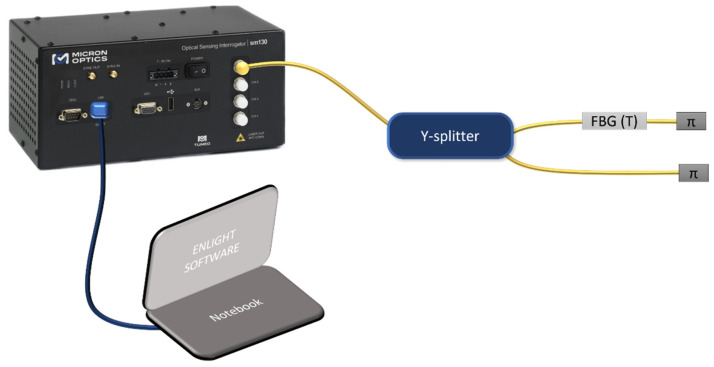
Experimental setup for the connection of 2 π probes on the same interrogation channel.

The only disadvantage is that it is impossible to have more than one sensor along the same optical fiber (hence, the same optical channel), but a solution may be given by connecting a 3 dB Y splitter in a fiber in order to connect two π probes on the same channel (Figure 4). In this way, when the resin lies at the end of the first probe, the reflected signal will be subjected to an attenuation of 3 dB, while when the second probe is also in contact with the resin, the signal will be totally attenuated.

### 2.2. T Sensor for Temperature Monitoring

There is a need for a temperature sensor which may co-exist with the π probe and the presence of the resin medium. Moreover, in the case of connection with the Y splitter, it is important that the two sensors do not affect each other during the measurement. An optical device that satisfies all the abovementioned requirements is the FBG sensor. This latter is realized within a fiber optic core due to refractive index modulation in a well-defined zone [18,19] with resonant behavior in its spectrum: at a certain wavelength (called the Bragg wavelength), the reflectivity is very high, reflecting the incoming spectrum in a narrow part. The Bragg wavelength can be chosen ad hoc, avoiding interference troubles if more than one FBG sensor is located in the same optical fiber. This device can be used as a sensor since both thermal and mechanical stresses are transduced in terms of shifts in Bragg wavelength from Equation (2).

(2) $$\Delta \lambda_{B}=S_{\epsilon }\epsilon +S_{T}\Delta T$$

where $S_{\epsilon }$ and $S_{T}$ are the strain and temperature sensitivity, respectively. Typical values at 1550 nm are 1.2 pm/μϵ and 10 pm/°C; with the commercial interrogation system, it is possible to sense 1 pm of shift in Bragg wavelength, that is 0.1 °C and 1 μϵ.

**Figure 5:**
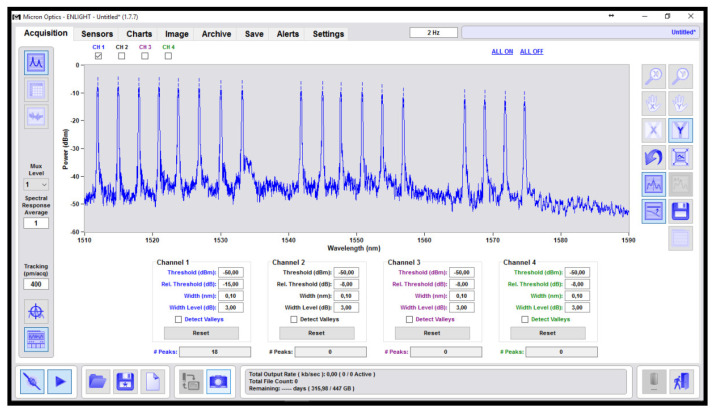
Spectrum of 18 FBGs used as temperature sensors.

For the case study, regarding the FBG characteristics, no specific requirements were needed. Hence, the FBG chosen were standard, with the following characteristics (Table 2) and spectrum (Figure 5).

## 3. Measurement Setup

**Figure 6:**
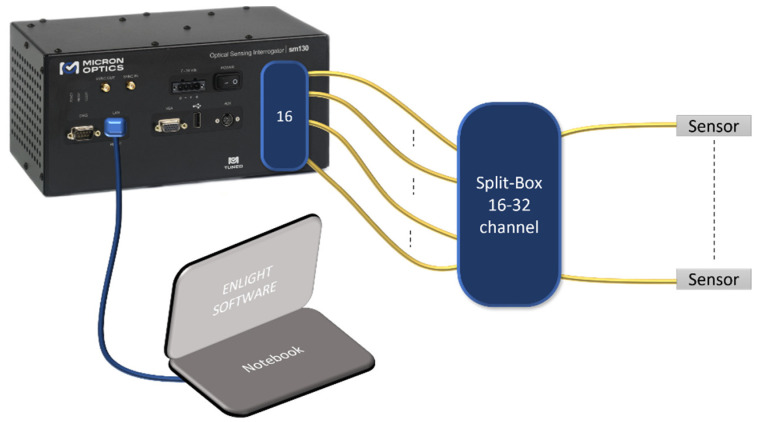
Sensing setup with the passive “split-box” to ensure the interrogation of 22 sensors.

Among many studies concerning the solutions to be implemented for FOS sensing (e.g., [20,21]), the abovementioned experimental test was carried out by employing a commercial system working both as an FOS interrogator and spectrometer: the Micron Optics sm225–800. With this piece of equipment, it was possible to monitor the shifts in Bragg wavelength in the FBG sensors (T) and to observe reflectivity changes at the fiber optic front-end in the presence of resin (π). The block scheme proposed in Figure 6 shows the Micron Optics Interrogator (MOI) with 16 output channels. Since 22 sensors were tested (18 T and 22 π), a splitter was needed in order to be able to simultaneously interrogate the comprehensive sensor network. The splitter was a passive “split-box”, composed by 16 fiber optic Y junctions with the ability to use up to 32 channels for monitoring purposes Regarding data monitoring, the MOI system used Enlight software, opportunely configured for sensor registration, saving and visualization. For data manipulation, MATLAB software was utilized for the elaboration and plotting.

### Test Preparation

The test panel that was sensorized was composed of one skin and four stringers, in which 18 FBG sensors were installed for temperature sensing, while 22 π probes were installed to detect the presence of resin. Since a single optical fiber can contain at least one FBG and one π probe, a sensor network is made up of several parallel optical fibers. For the considered structure, three different kinds of connections between the interrogator and the generic sensor were present. The generic optical fiber had to pass through a vacuum bag, an IN resin pipe and an OUT resin pipe. This enabled points of interest to be measured. In summary, the output modalities regarding the connections were as follows:

- A number of optical fibers inside the entering resin pipe, connected to $\Omega_{IN}$;
- A number of optical fibers inside the outgoing resin pipe, connected to $\Omega_{OUT}$;
- A number of optical fibers going through the sealed vacuum bag.

**Figure 7:**
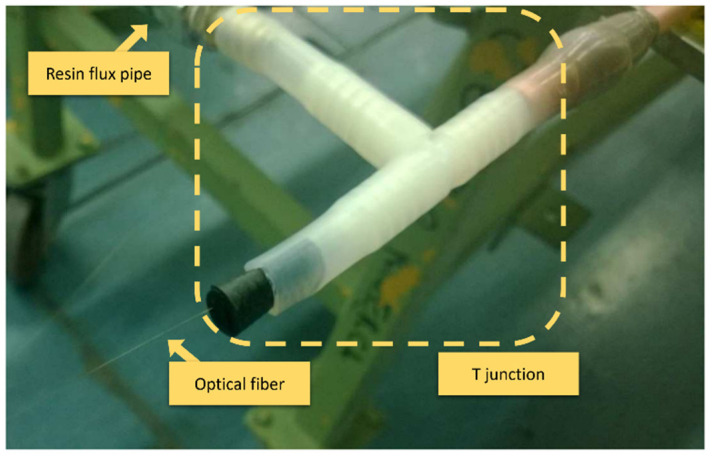
Fiber optic connected though T connector.

**Figure 8:**
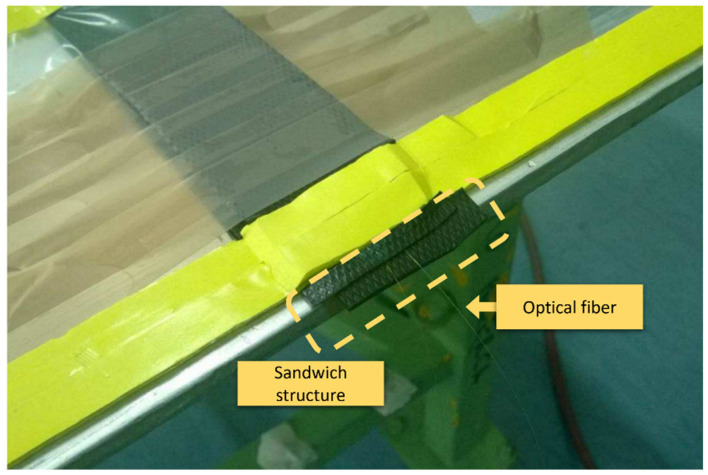
Two optical fibers installed inside and on the skin coming out through the sealed vacuum bag.

The connections related to $\Omega_{IN}$ and $\Omega_{OUT}$ both had to cross the liquid resin flux pipe in both the input and output direction. The best solution was to use a T junction, as showed in Figure 7. Regarding the connection that passed through the vacuum bag, the fiber was connected as linearly as possible, avoiding high bending that would induce optical signal losses. As shown in Figure 8, a sandwich structure was worked around the fiber path, ensuring a support and favoring a better pressure distribution during the vacuum condition.

**Figure 9:**
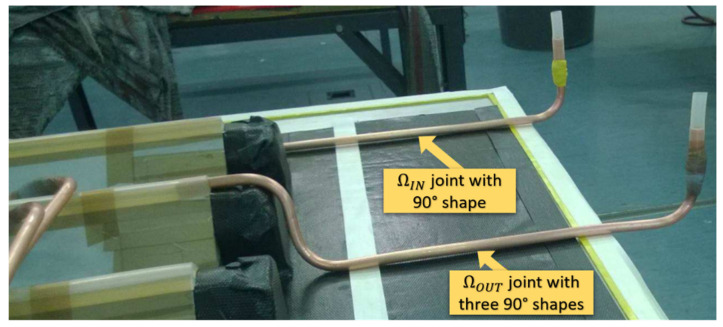
Copper pipe bending relative to $\Omega_{IN}$ and $\Omega_{OUT}$.

As depicted in Figure 9, a problem was created by the presence of many curvatures along the copper pipes (relative to $\Omega_{IN}$ and $\Omega_{OUT}$) for the right connection to the oven. This latter situation meant it was tricky for the π probes to be inserted, but it was achieved successfully without damaging the sensors.

**Figure 10:**
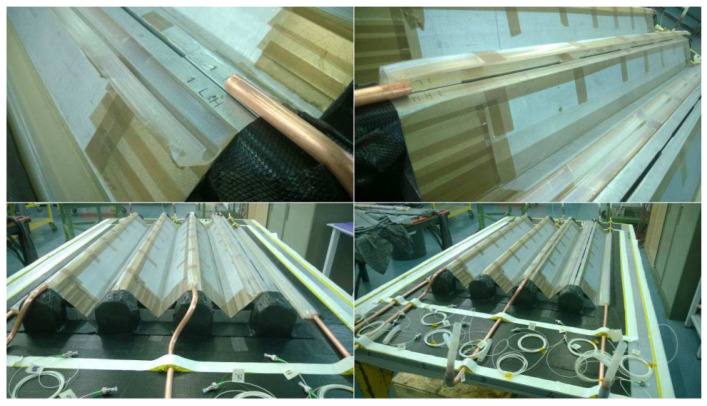
Stringer sensorization with $\Omega_{OUT}$ opened.

Concerning $\Omega_{OUT}$, on the stringer top, these were opened to ensure the correct FBG positioning. In Figure 10, multiple pictures show this installation process.

**Figure 11:**
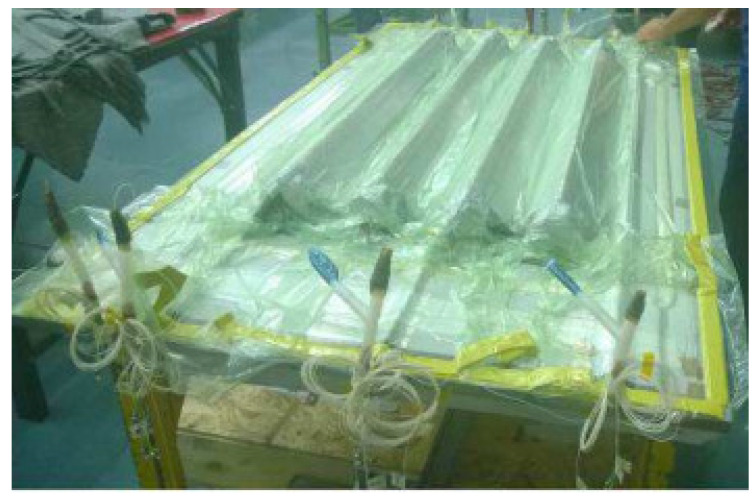
Final panel with sealed vacuum bag, ready for the infusion.

Finally, the fibers that came from the skin were routed along the outer edge in a straight line. These were placed on the sealant, which worked as a barrier for the resin. In Figure 11, the final panel is shown with the vacuum bag totally sealed.

## 4. Results

### 4.1. π Sensors Results

**Figure 12:**
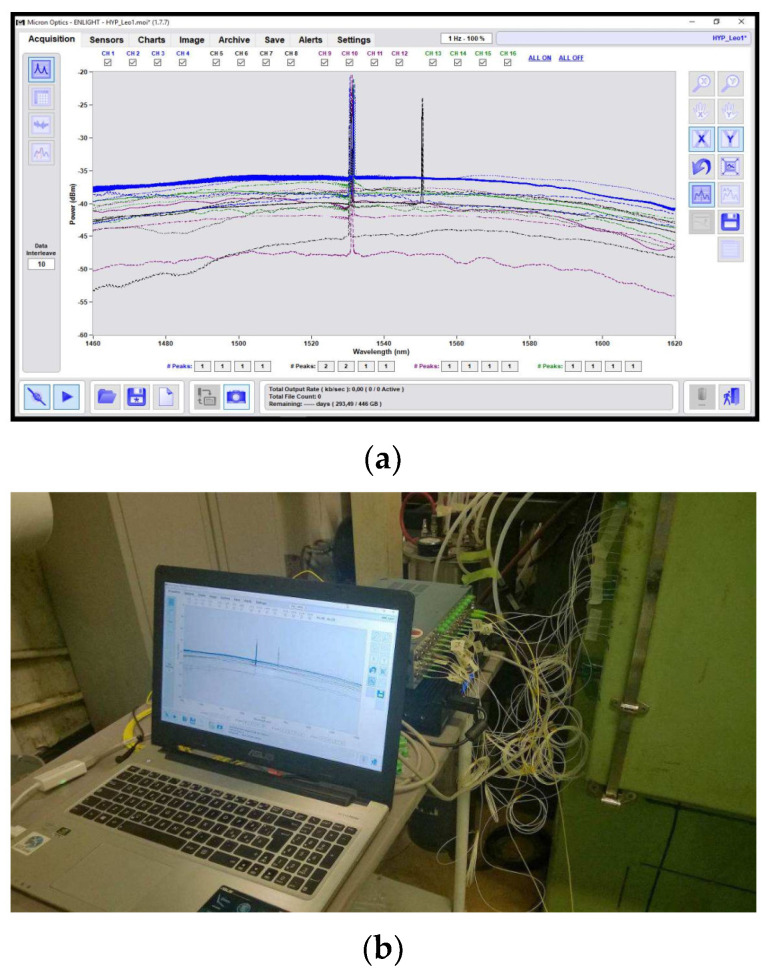
Acquisition system with the whole sensor network (**a**); magnified part of the Micron Optics Enlight software containing the whole sensor network spectrum (**b**).

**Figure 13:**
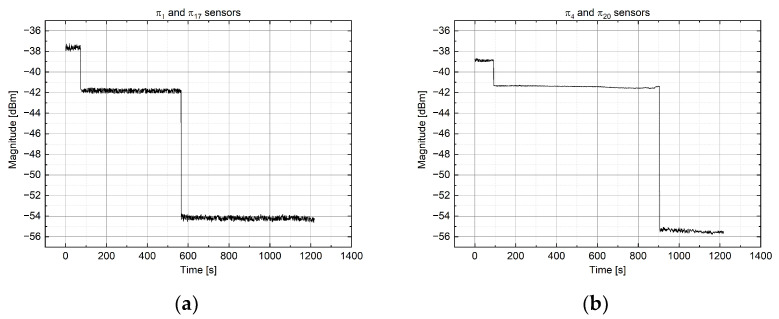
Resin presence on the sensor P1 and P17 (**a**) and P4 and P20 (**b**).

In Figure 12, the whole system is shown, arranged close to the oven. The fiber optic connections passed through the oven door, positioned one by one under a protective seal. The sensor network was completely readable from the MOI system, which is evidence of the optimal solutions adopted for the implementation. As an example, in Figure 13, two couples of π probes are displayed, in which the changes in the signal level caused by the resin presence are highlighted.

### 4.2. T Sensors Results

As it is widely known, FBG sensors are very sensitive to both temperature variation and changes in mechanical stress levels. For this reason, in order to ensure the correct measurement of just one effect (in this case study, the temperature), it is fundamental to make one sensor completely insensitive from another. In this work, an FBG in proximity of a fiber ending was mounted without any mechanical constraints; the fiber was simply affixed with a high-temperature tape. Although the preliminary test showed good results (wavelength interrogation was not influenced by strain), instable mechanical stress was evident from the experimental data, affecting the sensor thermal evolution. Actually, the majority of sensors were subjected to a high pressure applied from the vacuum bags to the materials, causing a strong compression on the fiber stretches containing FBGs. These phenomena led to many offsets over the sensors, shown by the mechanical stress which induced a shift in Bragg wavelength. This could be seen as, transducing the wavelength shift in temperature due to the thermal coefficient, the output value was different from the 120° expected (fixed inside the autoclave).

It is possible to examine the temperature at the instance of resin arrival in order to understand the related value and its gradient (the FBG became stabilized from the mechanical point of view when they were flooded by resin).

In the following, the temperature variation acquired with T1 and T4 sensors ($\Omega_{IN}$) is discussed, representative of what was found previously.

**Figure 14:**
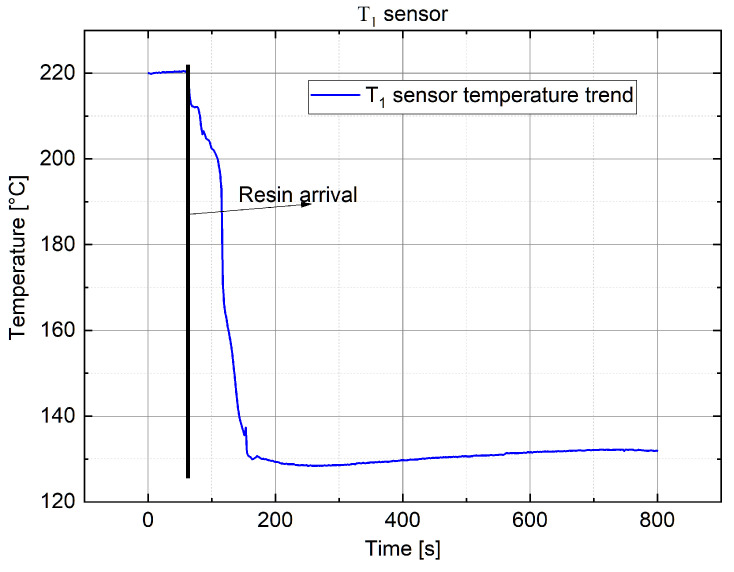
Temperature variation measured with FBG sensor T1.

**Figure 15:**
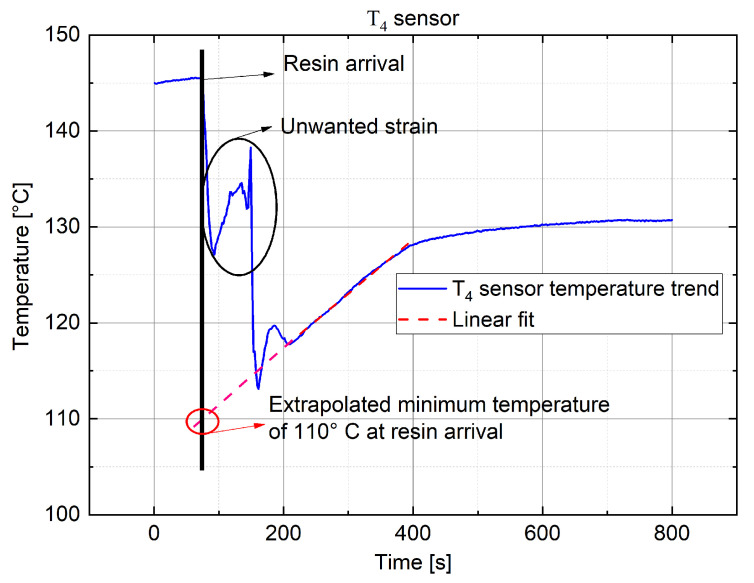
Temperature variation measured with FBG sensor T4 with an extrapolation to obtain the temperature value at the arrival of resin.

- In Figure 14, the temperature trend for sensor T1 is shown: in this case, the stress-induced offset is released with the arrival of resin. It is not possible to extrapolate a temperature estimation, but a temperature increment a few minutes after the arrival of resin can be noted, indicative of a change in the trend after a state of cooling.
- In Figure 15, the temperature trend for sensor T4 is shown: it is worth noting that, in this case, the offset released allowed us to accurately estimate the temperature value at the arrival of resin. This latter value was extrapolated by extending the curve, relative to the temperature increment, with its slope (in red) up to the intersection with the instance of the arrival of resin (stretch in black), which was provided by the π probe P4.

The information provided by sensor T4 allowed us to determine that the resin inside $\Omega_{IN}$ was at a higher temperature than the pre-heated one (90 °C) and, moreover, it tended to rapidly increase to the chamber temperature (118–120 °C). Furthermore, the sentence above implies that the resin came into the medium and was infused at a temperature close to 115 °C, aligned with the chamber temperature externally imposed. These considerations are confirmed by other sensors that, independently from the starting offset, did not give significative transients when in contact with the resin.

### 4.3. Agreement with Numerical Results

The filling times measured by FBG sensors, positioned at different locations of the panel (as shown in Figure 3), were in very good agreement with the results of the numerical simulation described in reference [16]. As an example, in the following table (Table 3), the comparison between the experimental and simulated data of the filling times measured on the top of the stringers is displayed. The described data represent the moment when the infusion of the panel ended. The numerical data were between 719 and 724 s, while the experimental data were between 700 and 764 s. This discrepancy (which involves an inaccuracy below 10%) might be due to an error on one or more parameters that affected the filling time. The main parameters were the vacuum level, the filling temperature and the vent configuration. Furthermore, among the parameters that affected the numerical model accuracy, it is worth mentioning the permeability data regarding the dry preforms which were determined experimentally, as reported in [16].

It should be underlined that a fault occurred in the measurement of the FBG sensor located on the fourth stringer because of a displacement.

## 5. Conclusions

In this paper, the LRI process was monitored for the purpose of its optimization and the validation of a numerical model. For this intent, a monitoring system based on fiber optic sensor technology was employed. The system was composed of a fiber optic sensor network in order to monitor both the temperature (18 points) and the resin arrival (22 points), carried out with a commercial MOI system suitable for static measurements (0.5 Hz as frequency sampling). Since the MOI is capable of monitoring 16 optical fibers, a passive split-box was made to allow use to investigate the whole network simultaneously.

The results obtained are satisfactory and provided qualitative and quantitative information useful for the aim of this study. The π optical probes, designed for the detection of the presence of resin, showed correct behavior, recognizing the resin presence in an unmistakable manner. Thanks to this good correlation, the numerical model developed in [16] was validated, allowing the Leonardo Sp.A. Aircraft Division to optimize the infusion strategy and the relevant bagging scheme for composite stiffened panels produced with LRI technology. Furthermore, this work revealed that the tested FOS network is a reliable and feasible system that can be implemented into industrialized infusion equipment for in-process inspection purposes. Regarding FBG sensors for temperature monitoring, a correlation between the shift in Bragg wavelength and the temperature was not found due to the presence of mechanical stress that affected many sensors. However, the extrapolated and post-processed monitoring data allowed us to estimate (in some cases) the temperature value at the arrival of resin, confirming the non-presence of transients as well as significative temperature lowering on the sensors.

## Figures and Tables

**Table 1:** Sensor distribution.

FOS Nr	Positioning	Monitoring
**4**	$2\;\mathrm{for}\;\mathrm{each}\;\Omega_{IN}$ at right and left of panel	T and π$(2\;\mathrm{for}\;\mathrm{each}\;\Omega_{IN}$)
8	$2\;\mathrm{for}\;\mathrm{each}\;\Omega_{OUT}$ on the top of each stringer	T and π$(2\;\mathrm{for}\;\mathrm{each}\;\Omega_{OUT}$)
2	On the skin, between stringer 1 and 2	T and π (2 locations)
2	On the skin, between stringer 2 and 3	T and π (2 locations)
4	$1\;\mathrm{for}\;\mathrm{each}\;\mathrm{output}\;\mathrm{resin}\;\mathrm{pipe}\;\mathrm{inside}\;\mathrm{the}\;\mathrm{oven}/\mathrm{autoclave}\;\mathrm{at}\;50\;\mathrm{cm}\;\mathrm{from}\;\Omega_{OUT}$	π (1 location per pipe)

**Table 2:** FBG characteristics.

Bragg grating length	10 mm
Maximum reflectivity	>90%
Full Width and Half Maximum (FWHM)	<0.3 nm
Side Lobe Suppression Ratio (SLSR)	15 dB

**Table 3:** Comparison between numerical and experimental results.

	Stringer Sensor Simulated Time (s)	Mean of Measured Time (s)
Stringer 1 (P9–P10)	719	764
Stringer 2 (P11–P12)	724	700
Stringer 3 (P12–P13)	724	705
Stringer 4 (P15–P16)	719	--

## Data Availability

Data underlying the results presented in this paper are not publicly available at this time but may be obtained from the authors upon reasonable request.
